# Exploring Age and Sex Differences in the Use of Cannabis Vaping Products: Results From the Canadian Cannabis Survey 2020–2023

**DOI:** 10.1111/dar.70155

**Published:** 2026-04-13

**Authors:** Fathima Fataar, Matthew J. Dann, Samantha Goodman, Hanan Abramovici

**Affiliations:** ^1^ Office of Cannabis Science and Surveillance Controlled Substances and Cannabis Branch, Health Canada Ottawa Canada; ^2^ School of Public Health Sciences University of Waterloo Waterloo Canada

**Keywords:** age groups, canada, cannabis, sex, vaping

## Abstract

**Introduction:**

Use of cannabis vaping products has been increasing in Canada. This study examined the prevalence and patterns of use of cannabis vaping products, including higher‐risk use, by sex and age.

**Methods:**

Data were from four cycles of the Canadian Cannabis Survey, an annual web‐based survey of people aged ≥ 16 years in Canada. Past 12‐month consumers of cannabis for non‐medical purposes were analysed (*n*_2020_ = 3405; *n*_2021_ = 2802; *n*_2022_ = 2801; *n*_2023_ = 2972). Cannabis vaping measures included frequency of use and co‐use with nicotine or tobacco. Logistic regression models tested differences in outcomes, overall and by sex and age.

**Results:**

Among past 12 month cannabis consumers, use of cannabis vaping products in the past 12 months increased from 22% (95% CI 20%–23%) in 2020 to 37% (95% CI 35%–39%) in 2023, with increases in all sex and age categories, most notably youth (16–19 years) whose use doubled from 33% (95% CI 27%–38%) to 66% (95% CI 60%–72%). Daily/almost daily use of cannabis vaping products also increased overall, from 2% (95% CI 1%–2%) in 2020 to 6% (95% CI 5%–7%) in 2023. There were few sex differences in patterns of use. The proportion of vaping product consumers classified as low risk increased from 13% (95% CI 10.3–16.6) in 2020 to 22% (95% CI 19.1–24.7) in 2023. The proportion classified as high risk also increased, while the proportion classified as moderate risk decreased.

**Discussion and Conclusions:**

Following legalization (2020 to 2023), there was a continued increase in the use of cannabis vaping products, especially among youth. Continued monitoring as well as additional public education efforts, especially related to potency and risks of vaping, are warranted.

## Key Point Summary

- Use of cannabis vaping products increased in Canada between 2020 and 2023.
- More vaping product consumers were categorized as low and high risk of cannabis‐related problems over time, and fewer as moderate risk.
- Continued monitoring and public education about vaping are warranted.

## 1 Introduction

Legalization and regulation of cannabis in Canada was implemented in two phases. The first phase, starting in October 2018, saw the legalisation, regulation, retail sale and distribution of dried cannabis, oral cannabis oils, seeds, plants and fresh cannabis. The second phase followed in October 2019 and introduced edibles, extracts (including vaping products) and topicals. Retail and online sales of these products began in December 2019 in some provinces [1].

While dried flower remains the most commonly purchased cannabis product from the legal market in Canada, inhaled extracts are the second most purchased product [2]. In contrast to smoking cannabis, where the smoke from combusted dried cannabis is inhaled, vaping methods typically heat liquid cannabis extracts to a point below combustion, and the ensuing aerosol is inhaled [3]. Vape pens or devices are often small and the vapour lacks the distinct cannabis smell, making their use more discrete and convenient [4, 5]. This makes cannabis vaping products more appealing to youth who may be trying to consume cannabis without detection [5, 6]. The concentration of the main psychoactive component of cannabis, delta‐9‐tetrahydrocannabinol (THC), is often much higher in cannabis vaping products (70%–90%) than in most dried flower products (up to ~30%) [7]. High THC concentrations have been associated with numerous health risks, including cannabinoid hyperemesis syndrome and various acute and chronic mental health disorders [8, 9]. Although the respiratory risks specifically associated with inhaling cannabis aerosols from vaping products may be lower than those from smoking cannabis [8], potential harms associated with cannabis vaping include oral health problems, impaired cognitive performance, seizures, and respiratory issues such as e‐cigarette or vaping‐associated lung injury, typically linked to vitamin E acetate present in unregulated vape products [3, 6]. There is currently little known about the long‐term effects of cannabis vaping on lung health [3, 4, 6].

The use of cannabis vaping products in Canada has been increasing steadily among cannabis consumers since 2018. A 2022 study using data from 2018 to 2020 found that use of cannabis vaping liquids increased from 21% in 2018 to 26% in 2020 among past 12‐month consumers. However, daily use, defined as use more than 5 days a week, remained relatively stable at 2% in 2018 and 3% in 2020 [10]. A more recent study examining trends in the use of cannabis products using data from the Canadian Cannabis Survey (CCS) reported an increase in past 12 month vape pen/cartridge use from 16% in 2018 to 33% in 2022 among past‐12 month cannabis consumers [11].

Furthermore, a substantial amount of research has explored the use of cannabis vape products among youth and young adults, including several reviews, although there is considerable variability in the outcomes reported [5, 10, 12, 13, 14, 15, 16]. In Canada, there has been a notable increase in cannabis vaping among both youth and young adults since surveillance efforts began around 2015 [6, 17, 18]. Estimates from national surveys since then suggest that almost three‐quarters of youth (grades 7 to 12) and half of post‐secondary students (aged 17–25) who consumed cannabis in the past 12 months reported using cannabis vaping products [18, 19]. This is concerning given that one of the main objectives of the *Cannabis Act* was to keep cannabis out of the hands of youth [1].

Existing literature examining sex differences in cannabis vaping suggests that among young people, males are more likely to use cannabis vaping products than females [6], whereas findings among adults have been mixed. In Canada, one study reported that from 2018 to 2019, adult males were more likely to consume cannabis vape products than females [14]. However, other research has found that among adult past 12‐month cannabis consumers in 2020, weekly or more frequent use of cannabis vaping liquids was similar for males and females, at approximately 8% [10]. Further research is required to monitor trends in prevalence of use by sex, as well as differences in frequency and other patterns of use.

Many consumers perceive cannabis vaping products to be better tasting, less harmful and more efficient at achieving their desired effects compared to traditional (i.e., smoked) forms of inhalation [6, 20]. This may result in an increased frequency of use compared to smoked forms of inhalation, especially among youth and young adults [21, 22]. This is particularly concerning because little is known about the long‐term effects of frequently using high‐THC vaping products [20, 21, 23], and because high frequency cannabis use in general has been associated with increased risk for cannabis‐related harms [21, 24, 25]. Moreover, many youth and young adults who vape cannabis also use other modes of administration [26, 27] and are more likely to consume nicotine and tobacco products which carry their own risks [6].

The current study had two primary objectives: (i) to examine trends in cannabis vaping in Canada from 2020 to 2023 overall and by sex and age; and (ii) to explore characteristics of use of cannabis vaping products and risk for cannabis‐related harms from use by sex and age. Given the high prevalence of cannabis vape product use, particularly among youth and young adults, and the limited literature characterising use following legalisation of cannabis vaping products, understanding patterns of use and the potential impact of use on different sub‐populations is important.

## 2 Methods

Data are from 4 years (2020–2023) of the CCS, which is an annual cross‐sectional study developed and implemented by the Government of Canada to monitor patterns of cannabis use and views on cannabis among people in Canada 16 years of age and older. Based on a quota sampling strategy by province/territory, age and sex groupings in Canada, the survey firm Advanis contacted people from a list of in‐service mobile and landline phone numbers. Potential respondents were told the survey was about cannabis use, and interested respondents were sent a link to the online survey by email or text message. Respondents provided informed consent prior to starting the survey and completed the survey in English or French. Respondents could skip any question they did not want to answer. A detailed description of the survey methodology is available online [28].

### 2.1 Measures

Demographic measures included age, sex and gender, race/ethnicity, education and income. Sex at birth rather than gender was used in analyses due to the small number of responses for other or unstated gender identity.

#### 2.1.1 Cannabis Vape Pen/Cartridge Use

Past 12 month vape pen/cartridge cannabis use was established with the question, “In the past 12 months, have you used the following cannabis products?… Cannabis vape pens/cartridges” (Yes/No/Don't know/Prefer not to answer). For the purposes of this study, ‘cannabis vaping products’ or ‘vaping products’ refers to the use of cannabis vape pens/cartridges. Because CCS only asks about ‘usual source’ of cannabis overall and not per product type, the legal status of vape products used by respondents (legal/illegal/unknown) could not be confirmed.

#### 2.1.2 Characteristics of Use for Past 12‐Month Cannabis Vape Pen/Cartridge Consumers

**Frequency of Use.** Among past 12 month cannabis consumers, the frequency of use of vaping products was measured with the question, “In the past 12 months, how often have you used the following products for non‐medical purposes? Cannabis vape pens/cartridges.” Response options were recoded to 4 categories: Less than monthly/Monthly (1–3 days/month)/Weekly (1–2 days/week)/Daily or almost daily (5 or more days/week).

**Exclusive Use of Vape Pens/Cartridges.** Respondents use was classified as ‘Exclusive use’ if vape pen/cartridges were the only cannabis product consumed in the past 12 months, and ‘Non‐exclusive use’ if they reported using any other cannabis product in addition to vape pen/cartridges.

**Co‐Use With Tobacco or E‐Cigarettes with Nicotine.** Respondents were asked how often they mixed or consumed tobacco or e‐cigarettes at the same time as cannabis in the past 12 months. This was recoded into two groups: Never/Rarely or Always/Often/Sometimes.

**World Health Organization Alcohol, Smoking and Substance Use Involvement Screening Test (ASSIST).** This pre‐established measure was used to assess the level of risk for developing health and other problems from cannabis use [29]. Five questions in the areas of frequency of use, urge to use, interference with responsibilities, difficulty cutting down and concern expressed by others were used to generate an overall score. Total scores range from 0 to 39 and are categorised into three groups: low risk (0–4)/moderate risk (5–26)/high risk (> 26). For this study, a binary response was used: Low/Moderate risk and High risk for cannabis‐related harms. Full survey questions for all variables are presented in Data S1.

### 2.2 Data Analysis

Data from the CCS were weighted based on age, sex and region (province/territory) using Canadian census estimates to more accurately reflect the general population. After excluding four participants who were missing age, six who were missing both sex and gender, 1970 who consumed cannabis for exclusively medical purposes, and 584 with missing data on use of vape pens/cartridges, analyses were conducted on a sub‐sample of 11,980 respondents who had consumed cannabis for exclusively non‐medical or both non‐medical and medical purposes in the past 12 months (*n*_2020_ = 3405; *n*_2021_ = 2802; *n*_2022_ = 2801; *n*_2023_ = 2972).

Logistic regression models were used to examine changes in past 12‐month and daily/almost daily use of cannabis vaping products over time, as well as to examine differences by sex and age. The study period spanned four timepoints (2020, 2021, 2022 and 2023). Furthermore, logistic regression models were used to compare characteristics of use (frequency of use, exclusive vaping and co‐use with nicotine/tobacco) and risk for cannabis‐related problems among those who consumed cannabis vape pen/cartridges by sex and age. Models were adjusted for ethnicity, education level, household income and survey year. Observations with missing data for covariates were excluded from models on a listwise basis.

A 3‐category age variable (16–19, 20–24, 25+ years) was used for the above analyses. To ensure that patterns of use among older adults were not being obscured, sensitivity analyses were conducted to test changes in prevalence of vaping over the study period using a 6‐category variable (16–19, 20–24, 25–34, 35–44, 45–54, 55+ years).

All analyses were conducted in SAS 9.4 using survey‐routine procedures and survey weights. Adjusted odds ratios and 95% confidence intervals are shown. Estimates are weighted unless specified otherwise. This analysis received ethics clearance from the Health Canada—Public Health Agency of Canada Research Ethics Board.

## 3 Results

Approximately three‐quarters of respondents were white, close to half were female, and mean age was 38.6 years (37.7 in 2020, 38.7 in 2021, 39.2 in 2022, 38.8 in 2023). Remaining sample characteristics are shown in Table S2.

### 3.1 Past 12‐Month Use of Cannabis Vaping Products

**FIGURE 1:**
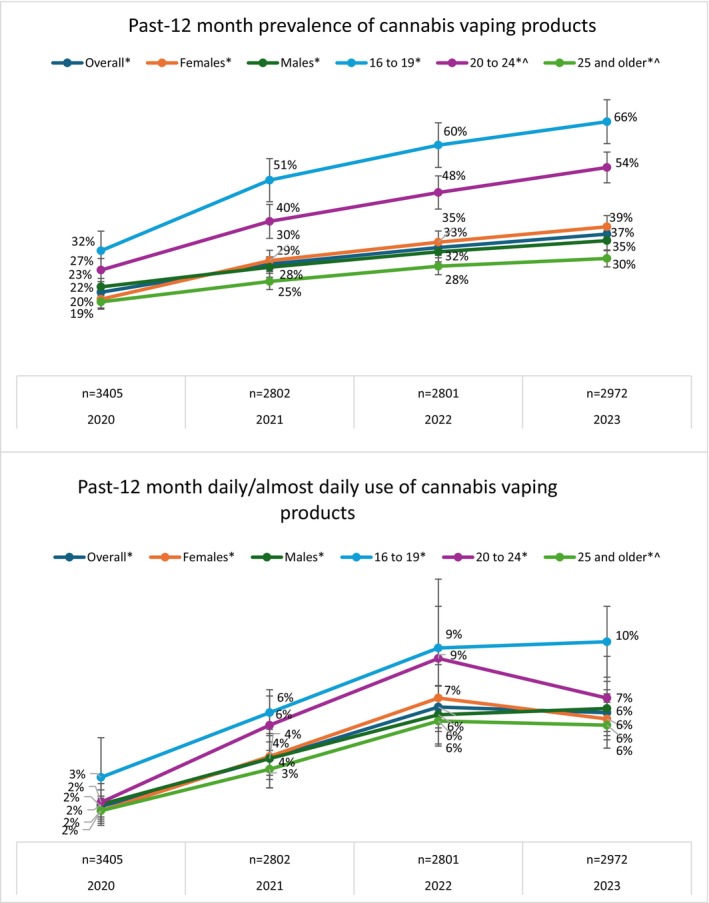
Use of cannabis vaping products among past 12‐month cannabis consumers overall and by sex and age 2020–2023 (*n*_2020_ = 3405; *n*_2021_ = 2802; *n*_2022_ = 2801; *n*_2023_ = 2972). *Significant difference between 2020 and 2023 after adjusting for sex, age, ethnicity, income and education (*p* < 0.05); ^Significantly different from 16 to 19 year olds (reference group).

As Figure 1 shows, among those who consumed cannabis products in the past 12 months, use of vaping products increased from 22% in 2020 to 37% in 2023. Overall, there was no difference in the prevalence of use of vaping products among males and females. Use of cannabis vaping products increased in all age groups, with the greatest increase among those aged 16–19 and 20–24 whose use increased by 33% and 27%, respectively, from 2020 to 2023. Youth aged 16 to 19 had greater odds of using cannabis vaping products than respondents in older age categories (see regression results; Table S3).

Sensitivity analyses using the 6‐category age variable indicated the same pattern of results among adults 25 and older, although the magnitude of change was smaller. Use of vaping products increased significantly in all adult age groups: by 15% among 25–34 year‐olds, and by 9%–10% among 35–44, 45–54 and 55+ year‐olds. Again, youth aged 16 to 19 had greater odds of using cannabis vaping products than respondents in all older age categories. Point estimates and regression results are shown in Table S3. In 2020 and 2021, cell counts were too small to release estimates of daily/almost daily (DAD) use of vaping products among the three oldest age categories. Therefore, the 3‐category age variable (16–19, 20–24, 25+) was retained for subsequent analyses.

DAD use of vaping products among past 12‐month cannabis consumers increased from 2% in 2020 to 6% in 2023 (Figure 1). Increases from 2020 to 2023 were observed in all subgroups, with 3–4 times the odds of using DAD in 2023 vs. 2020 in both sexes and all three age groups.

### 3.2 Patterns of Use Among Cannabis Vaping Product Consumers

#### 3.2.1 Frequency of Use

Tables 1 and 2 present results on the patterns of use for cannabis vaping products by sex and age, respectively. Among consumers who had used vaping products in the past 12 months, the majority of respondents consumed vaping products less than monthly. While approximately 8% of both male and female vaping product consumers consumed cannabis vaping products DAD in 2020, this increased to 18% for males and 15% for females in 2023. In 2020, 9% of 16–19‐year‐olds, 7% of 20–24‐year‐olds, and 8% of adults 25 and older consumed vaping products DAD; this increased to 15%, 13% and 19%, respectively, in 2023. There were no notable differences in frequency of use between 16 and 19‐year‐olds and 20–24 year‐olds; however, in adjusted models, both groups were less likely than those 25 and older to consume DAD. In addition, those 20–24 were less likely to consume weekly than those 25 and older (Table 3, Model 1).

**TABLE 1:** Characteristics of use of vape pen/cartridge products among past 12‐month vaping product consumers by sex (2020–2023)^a^.

	2020		2021		2022		2023	
	Females	Males	Females	Males	Females	Males	Females	Males
	*n* = 310	*n* = 461	*n* = 425	*n* = 433	*n* = 471	*n* = 518	*n* = 563	*n* = 599
	% (95% CI)	% (95% CI)	% (95% CI)	% (95% CI)	% (95% CI)	% (95% CI)	% (95% CI)	% (95% CI)
Frequency of use								
Less than monthly	40.6 (34.3, 46.9)	39.7 (34.5, 45.0)	32.2 (27.2, 37.3)	24.6 (29.2, 39.1)	34.1 (29.2, 39.1)	31.4 (26.8, 36.1)	34.7 (30.2, 39.1)	30.3 (26.2, 34.3)
Monthly	33.6 (27.6, 39.5)	30.7 (25.9, 35.5)	32.1 (27.0, 37.2)	31.6 (26.7, 36.5)	24.4 (20.1, 28.7)	25.3 (21.0, 29.6)	24.7 (20.7, 28.7)	28.6 (24.5, 32.7)
Weekly	18.0 (13.2, 22.8)	21.6 (17.4, 25.8)	21.8 (17.4, 26.2)	29.2 (24.4, 34.0)	21.4 (17.3, 25.6)	24.0 (20.0, 28.1)	25.4 (21.4, 29.5)	22.9 (19.2, 26.5)
Daily/Almost daily	7.8* (4.4, 11.2)	8.0* (5.1, 10.9)	13.9 (10.2, 17.6)	14.6 (10.8, 18.4)	20.0 (15.9, 24.2)	19.3 (15.3, 23.3)	15.2 (11.8, 18.6)	18.3 (14.9, 21.7)
Exclusive vaping								
Vape pen/cartridge only	10.2 (6.1, 14.3)	7.6 (4.7, 10.6)	5.4 (3.0, 7.8)	6.2 (3.6, 8.8)	9.4 (6.4, 12.5)	8.4 (5.6, 11.1)	9.9 (7.1, 12.8)	10.2 (7.5, 12.9)
Vape and at least one other cannabis product	89.8 (85.7, 93.9)	92.4 (89.4, 95.3)	94.6 (92.2, 97.0)	93.8 (91.2, 96.4)	90.6 (87.5, 93.6)	91.6 (88.9, 94.4)	90.1 (87.2, 92.9)	89.8 (87.1, 92.5)
Use with tobacco or e‐cigarette containing nicotine								
Never/Rarely	64.5 (58.6, 70.4)	64.4 (59.5, 69.3)	69.4 (64.6, 74.2)	63.4 (58.4, 68.4)	70.0 (65.4, 74.7)	63.5 (58.8, 68.3)	63.7 (59.2, 68.2)	64.2 (60.0, 68.4)
Always/Often/Sometimes	35.5 (29.6, 41.4)	35.6 (30.7, 40.5)	30.6 (25.8, 35.4)	36.6 (31.6, 41.6)	30.0 (25.3, 34.6)	36.5 (31.7, 41.2)	36.3 (31.8, 40.8)	35.8 (31.6, 40.0)

*Note:* All estimates are weighted.

Abbreviation: CI, confidence interval.

^a^ Only includes past 12‐month consumers who reported using vape pens/cartridges.

* Small sample size; interpret with caution.

**TABLE 2:** Characteristics of use of vape pen/cartridge products among past 12‐month vaping product consumers by age (2020–2023)^a^.

	2020			2021			2022			2023		
	16–19	20–24	25+	16–19	20–24	25+	16–19	20–24	25+	16–19	20–24	25+
	*n* = 118	*n* = 248	*n* = 405	*n* = 174	*n* = 211	*n* = 473	*n* = 178	*n* = 263	*n* = 548	*n* = 199	*n* = 338	*n* = 625
	% (95% CI)	% (95% CI)	% (95% CI)	% (95% CI)	% (95% CI)	% (95% CI)	% (95% CI)	% (95% CI)	% (95% CI)	% (95% CI)	% (95% CI)	% (95% CI)
Frequency of use												
Less than monthly	38.4 (28.9, 47.9)	40.3 (33.9, 46.8)	40.4 (35.0, 45.7)	33.7 (26.2, 41.2)	30.1 (23.8, 36.5)	26.2 (21.7, 30.7)	32.7 (25.6, 40.0)	29.3 (23.6, 35.0)	33.7 (29.2, 38.3)	31.5 (24.7, 38.4)	36.7 (31.3, 42.1)	31.3 (27.2, 35.4)
Monthly	32.4 (23.4, 41.4)	36.6 (30.4, 42.9)	30.4 (25.4, 35.3)	26.3 (19.6, 33.1)	32.9 (26.2, 39.6)	32.7 (27.9, 37.6)	27.5 (20.8, 34.3)	31.1 (25.2, 37.0)	22.5 (18.5, 26.4)	30.7 (23.9, 37.6)	26.3 (21.3, 31.2)	25.9 (22.0, 29.7)
Weekly	19.8* (12.1, 27.4)	16.1 (11.3, 20.8)	21.3 (17.1, 25.6)	27.6 (20.5, 34.7)	23.0 (16.9, 29.0)	26.2 (21.8, 30.7)	24.2 (17.7, 30.7)	21.0 (15.9, 26.2)	23.0 (19.2, 26.9)	23.1 (17.0, 29.2)	24.3 (19.5, 29.1)	24.3 (20.6, 28.0)
Daily/Almost daily	9.4* (3.7, 15.2)	6.9* (3.6, 10.2)	7.9* (5.0, 10.8)	12.4* (7.3–17.5)	14.0* (9.1, 18.9)	14.8 (11.2, 18.4)	15.5* (10.1, 20.9)	18.6 (13.6, 23.5)	20.8 (16.9, 24.7)	14.6* (9.4, 19.8)	12.8 (9.2, 16.4)	18.6 (15.2, 21.9)
Exclusive vaping												
Vape pen/cartridge only	7.6^#^ (2.1, 13.0)	4.0* (1.5, 6.6)	10.2 (6.9, 13.5)	6.6* (2.6, 10.7)	4.4* (1.6, 7.2)	6.2* (3.7, 8.6)	10.6* (5.8, 15.4)	8.4* (5.0, 11.9)	8.7 (5.9, 11.4)	13.7* (8.6, 18.8)	6.8* (3.9, 9.6)	10.2 (3.9, 9.6)
Vape and at least one other cannabis product	92.4 (87.0, 97.9)	96.0 (93.4, 98.5)	89.8 (86.5, 93.1)	93.4 (89.3–97.4)	95.6 (92.8, 98.4)	93.8 (91.4, 96.3)	89.4 (84.6, 94.2)	91.6 (88.1, 95.0)	91.3 (88.6, 94.1)	86.3 (8.6, 18.8)	93.2 (90.4, 96.1)	89.8 (90.4, 96.1)
Use with nicotine or tobacco or e‐cigarette containing nicotine												
Never/Rarely	40.3 (30.8, 49.9)	54.4 (48.0, 60.9)	72.3 (67.6, 77.1)	43.7 (35.9, 51.5)	61.6 (54.6, 68.5)	72.7 (68.2, 77.1)	50.2 (42.5, 57.8)	57.8 (51.4, 64.1)	72.2 (68.0, 76.6)	47.2 (39.9, 54.6)	54.6 (49.0, 60.1)	70.9 (66.9, 74.9)
Always/Often/Sometimes	59.7 (50.1, 69.2)	45.6 (39.1, 52.0)	27.7 (22.9, 32.4)	56.3 (48.5, 64.1)	38.4 (31.5, 45.4)	27.3 (22.9, 31.8)	49.8 (42.2, 57.5)	42.2 (35.9, 48.6)	27.7 (23.4, 32.0)	52.8 (45.4, 60.1)	45.4 (39.9, 51.0)	29.1 (25.1, 33.1)

*Note:* All estimates are weighted.

Abbreviation: CI, confidence interval.

^a^ Only includes past 12 month consumers who reported using vape pens/cartridges.

* High sampling variability; interpret with caution.

**TABLE 3:** Logistic regression models comparing characteristics of use of vape pen/cartridge products among past 12 month consumers by age and sex (2020–2023)^a^.

	Male vs female (ref)^b^	6–19 vs. 25+ (ref)^c^	20–24 vs 25+ (ref)	16–19 vs 20–24 (ref)
	AOR, 95% CI	AOR, 95% CI	AOR, 95% CI	AOR, 95% CI
Outcome^d^				
Model 1: Frequency of use (*n* = 3748)				
Less than monthly	Reference*	Reference	Reference	Reference
Monthly	1.16 (0.96, 1.41), *p* = 0.124	0.77 (0.57, 1.04), *p* = 0.085	0.95 (0.76, 1.17), *p* = 0.614	0.81 (0.61, 1.08), *p* = 0.159
Weekly	1.24 (1.02, 1.51), *p* = 0.035	0.83 (0.61, 1.14), *p* = 0.250	**0.79 (0.62, 0.99), *p* = 0.040**	1.06 (0.78, 1.45), *p* = 0.712
Daily/Almost daily	1.17 (0.92,1.49), *p* = 0.200	**0.45 (0.31, 0.66), *p* < 0.001**	**0.60 (0.45, 0.79), *p* < 0.001**	0.76 (0.53, 1.10), *p* = 0.143
Model 2: Number of cannabis products (*n* = 3780)				
Vape pen/cartridge only	Reference*	Reference	Reference	Reference
Vape and other cannabis product	1.06 (0.81, 1.60), *p* = 0.674	0.71 (0.47, 1.06), *p* = 0.89	1.36 (0.98, 1.88), *p* = 0.069	**0.52 (0.34, 0.80), *p* = 0.003**
Model 3: Use with tobacco or e‐cigarette containing nicotine (*n* = 3738)				
Never/Rarely	Reference	Reference	Reference	Reference
Always/Often/Sometimes	1.17 (1.00, 1.38), *p* = 0.048	**2.02 (1.58, 2.58), *p* < 0.001**	**1.61 (1.34, 1.93), *p* < 0.001**	1.26 (1.00, 1.59), *p* = 0.055

*Note:* Bolded outcomes indicate significant differences (*p* < 0.05).

Abbreviations: AOR, adjusted odds ratios; CI, confidence interval.

^a^ Only includes past 12‐month consumers who reported using vape pens/cartridges.

^b^ Models for sex comparisons are adjusted for age, education, income, ethnicity and survey year (2020, 2021, 2022, 2023).

^c^ Models for age comparisons are adjusted for sex, education, income, ethnicity and survey year (2020, 2021, 2022, 2023).

^d^ Differences in sample size reflect differences in missing data for the outcome variable.

* Type 3 analysis of effects for sex and the outcome were not significant (*p* > 0.05).

#### 3.2.2 Exclusive Use of Cannabis Vaping Products

Almost 90% of past 12‐month cannabis vaping product consumers also reported using other cannabis products, such as dried flower, edibles or concentrates (e.g., wax, shatter) (Table 1). While there were no differences by sex, the odds of using more than one cannabis product compared to exclusive vape use were lower among youth aged 16–19 than those aged 20–24 (Table 3, Model 2).

#### 3.2.3 Co‐Use of Tobacco and Nicotine

Approximately a third of both male and female vaping product consumers reported using tobacco or nicotine in combination with cannabis vaping products sometimes/often/always in the past 12 months. Adults 25 and older were less likely to use tobacco or nicotine in combination with vaping products than younger respondents (Table 3, Model 3). For example, in 2023, 53% of 16–19 year‐olds and 45% of 20–24 year‐olds reported combining tobacco or nicotine when using a vaping product at least sometimes, compared to only 29% of those 25 and older (Table 2).

### 3.3 Use of Cannabis Vaping Products and Cannabis‐Related Problems

**FIGURE 2:**
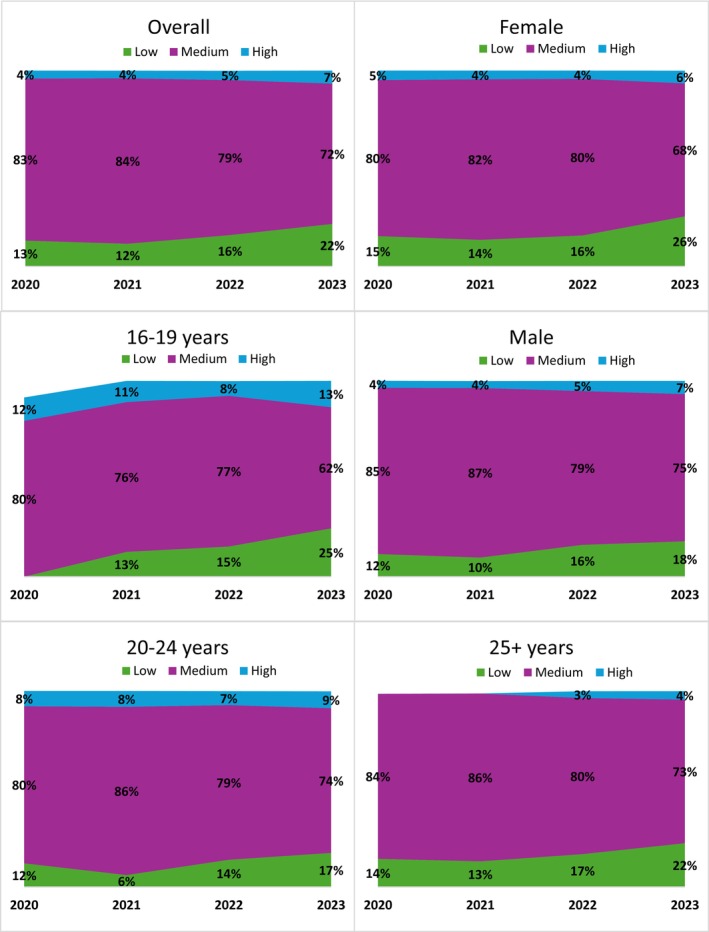
Proportion of cannabis vaping product consumers in each ASSIST risk category for cannabis‐related problems, 2020–2023 (*n* = 3450). Note: Estimates for ‘low’ risk in age 16–19 in 2020 and for ‘high’ risk in age 25+ in 2020 and 2021 were suppressed due to small cell counts and high sampling variability. The majority of estimates for the ‘low’ and ‘high’ risk groups have moderate sampling variability and should be interpreted with caution.

Overall, most respondents who consumed cannabis vaping products were classified as having ‘moderate’ risk for cannabis‐related problems. There were higher odds of vaping product consumers being classified as ‘low’ risk for cannabis‐related problems in 2023 versus 2019 (adjusted odds ratio 1.79 (1.29, 2.47), *p* < 0.001). There was no difference between 2020 and 2021 or 2022 (*p* > 0.05 for all). Regression modelling was not conducted for odds of being classified as ‘high’ risk on ASSIST, or stratified by age and sex groups due to moderate or high sampling variability for the ‘low’ and ‘high’ risk categories. However, as shown in Figure 2, in all age and sex groups, the proportion of respondents in the ‘moderate’ risk group (i.e., ‘brief intervention needed’) decreased over time from 83% to 72%, while the proportions in both the ‘low’ and ‘high’ risk groups increased. Youth aged 16–19 and 20–24 had the greatest proportion of vaping product consumers categorised as ‘high risk’, at 13% and 9%, respectively.^1^

## 4 Discussion

Overall, in Canada there was a substantial increase in the use of cannabis vaping products from 2020 to 2023. This is consistent with previous research showing recent increases in the use of vaping products in Canada [10, 11, 18]. CCS results also indicate changes in the use of other cannabis products. For example, cannabis edibles and beverages increased by 5% and 13%, respectively, with 54% of consumers using edibles and 19% using beverages in 2023. This was paralleled by a 14% decrease in the use of dried flower, with 60% of consumers using dried flower in 2023. Nevertheless, the overall prevalence of cannabis use remained relatively stable, ranging from 25% to 27% over the study period [30]. These data suggest a stabilisation of overall cannabis use in the post‐legalisation era, but a shift from traditional (dried flower) to alternative product forms.

While overall cannabis use is generally higher among males, we found that among cannabis users, similar proportions of males and females consumed cannabis vaping products. Prior research has found greater stigma around cannabis use in general for females [31]. It is possible that certain characteristics of vaping products (e.g., discreet, less odour) may be more appealing to females than conventional dried flower products. There was considerable variability in vaping product use among different age groups. From 2020 onward, there was a consistent pattern of a higher past 12‐month prevalence among young people aged 16–19 and 20–24 compared to adults 25 and older (Figure 1). In addition, the odds of cannabis vaping product use in 2023 were four times greater for 16–19 year olds and three times greater for 20–24 year olds compared to 2020. This aligns with previous literature [10, 32]. It also supports the need for additional public education or other measures/approaches directed at youth and young people regarding potential harms associated with using cannabis vaping products and how to reduce them.

Similar to past 12‐month use, DAD use of cannabis vaping products among past 12‐month consumers of all age and sex groups was substantially higher in 2023 than in 2020. Research shows that more frequent cannabis use increases the risk for cannabis use disorder [25], and cannabis vaping products tend to contain high concentrations of THC, increasing the risk for certain cannabis‐related harms [3, 33]. Interestingly, consumers of cannabis vaping products who were 25 and older were more likely to use DAD than youth and young adults. The current work presents overall prevalence of DAD vaping in the population, at 6% in 2023. However, CCS results also show that *among* consumers who report using each product type in 2023, DAD use of edibles (5%) and beverages (2%) is much lower than DAD use of vape pens/cartridges (18%) or dried flower (31%) [30]. So, although growing numbers of consumers report using edibles and beverages, frequent use remains more common for inhaled products. Further research with consumers of cannabis vaping products is needed to understand the factors underlying different patterns of use, and continued monitoring of frequency of use for cannabis vaping products is imperative.

Across all sex and age categories, most vaping consumers reported using at least one other cannabis product in addition to a vaping product. This is not surprising given that use of dried/flower and edible cannabis products are still more prevalent than use of vaping products in Canada [10, 12]. Given the variety of products that are currently available through the legal and illegal markets in Canada, and the differential effects and concentrations offered by different products, consumers may be choosing products based on the experience they are seeking or the occasion (e.g., social versus regular use).

Approximately half of youth using vaping products reported using nicotine or tobacco at the same time as cannabis sometimes/often/always, and both youth and young adults were more likely to co‐use than adults 25 and older. Still, close to a third of those aged 25 or older who consumed cannabis vaping products in the past 12 months reported co‐use of nicotine/tobacco products. These findings are consistent with research from the US which has found that among young people and adults, nicotine and tobacco use are associated with cannabis vaping [6, 34, 35]. The current study is unique in that it was able to examine simultaneous co‐use in Canada among both young people and adults over 25. Although the research is limited, one of the primary reasons people report simultaneous co‐use of cannabis and nicotine vaping products is enhancement of intoxicating effects [36, 37]. However, using nicotine/tobacco and cannabis together has been associated with increased risk for more severe withdrawal symptoms, as well as psychiatric and psychosocial issues, and co‐use can make cessation of either substance more difficult [3, 38]. Programs have been developed to promote smoking cessation, as well as cessation programs which target nicotine vaping among youth [39, 40]. However, education around co‐use and cessation programs which address cannabis and nicotine or tobacco co‐use for all age groups are warranted.

The ASSIST measure allows insight into the potential for cannabis‐related harms. The current study found that over time, there were higher proportions of vaping product consumers classified as ‘low’ risk, increasing from 13% to 22% over the study period. At the same time, in 2023, 13% of cannabis vaping product consumers 16–19 and approximately 9% of those aged 20–24 were classified as ‘high risk’ for cannabis‐related harms compared to 4% of those 25 and older. The higher percentage of young respondents classified as ‘high risk’ is of concern, as there is potential for long‐term impacts on health, relationships, and well‐being [41]. The ASSIST measure considers various areas of impact on one's life, including their relationships and ability to meet expectations, and individual questions were not explored in the current study. Therefore, it is unclear where the differences lie, and which areas may be impacted differently for younger people. Long‐term studies with a larger sample of cannabis vaping product consumers, including more young people, would allow further exploration of changes in outcomes over time as well as which factors are associated with higher risk scores. In addition, future research should examine if these findings are consistent across different measures for problematic cannabis use.

### 4.1 Limitations

This study is subject to limitations. As the data collected are based on self‐reports, social desirability and recall bias may influence estimates. In addition, self‐reported data rely on interpretation of the question and response options by the respondent. For example, some of the measures used in the current study included response options which ranged from never to always, and there is individual variation in interpretation of the responses ‘often’, ‘sometimes’, and ‘rarely’ which may impact results. In addition, ASSIST was used to assess ‘higher risk’ cannabis use because it was available in the 2020–2023 CCS survey cycles. The ASSIST relies heavily on frequency of use as a measure of problematic use (one reason that exclusive medical consumers were excluded from the analysis). ASSIST also has a large range for the moderate risk category (scores of 5–25), and those at the higher end of that range may actually be considered at ‘high risk’ for cannabis‐related harms. Using other measures such as the Severity of Dependence Score (added to CCS in 2023) or the Cannabis Use Disorder Identification Test may have led to different conclusions regarding subgroup differences or changes in ‘higher risk’ cannabis use. As the data are cross‐sectional, it is not possible to identify changes at the individual level, so any associations or trends reported are at the population level. The study also examined co‐use of cannabis with nicotine/tobacco. However, it did not differentiate between co‐use of cannabis plus nicotine and co‐use of cannabis plus tobacco, which may pose differing levels of health risk. Finally, this study coincided with the COVID‐19 pandemic, which led to increases in substance use. However, as we observed increases in cannabis vaping in 2023 compared to the height of the pandemic (2020–2021), it seems that vaping increased above and beyond the effects of the pandemic.

## 5 Conclusions

Past 12‐month and DAD cannabis vaping have increased since 2020, particularly among young people who use cannabis. Other patterns of use remained relatively consistent among vaping product consumers, including exclusive vaping and co‐use with tobacco or nicotine products. While cannabis vaping products may carry less respiratory risk than smoked cannabis, there is concern about appeal to young people and health risks associated with higher THC exposure. Continued monitoring of vaping product use and associated harms is warranted. Moreover, young people should be educated on the risks of using vape products. Communication should focus on risks related to potency, frequent use, and co‐use of nicotine and cannabis products. Longitudinal studies on the long‐term effects of cannabis vaping on health outcomes are needed.

## Author Contributions

Each author certifies that their contribution to this work meets the standards of the International Committee of Medical Journal Editors.

## Funding

This work was supported by Health Canada.

## Conflicts of Interest

This study was funded by Health Canada. The authors have no interests to declare.

## Supporting information


**Data S1:** Survey Measures.

**Table S2:** Sample characteristics of past 12‐month cannabis consumers aged 16 years and older, Canadian Cannabis Survey, 2020–2023.

**Table S3:** Use of cannabis vaping products among past 12‐month cannabis consumers overall and by sex and age, 2020–2023.

## Data Availability

The 2020 to 2022 Canadian Cannabis Survey (CCS) data are available online at: https://open.canada.ca/data/en/dataset/b65cb40c‐10e8‐4332‐ae4e‐0f1cc134f53f. The 2023 CCS data are available online at: https://open.canada.ca/data/en/dataset/6c240c79‐c857‐4fd4‐bbd6‐b1fd7f04fdbc.

## Endnotes

^1^
Estimates of ‘high risk’ use should be interpreted with caution due to moderate sampling variability.
