# Supplementary material for: Exploring Age and Sex Differences in the Use of Cannabis Vaping Products: Results From the Canadian Cannabis Survey 2020–2023
Source: Drug Alcohol Rev. 2026 Apr 13;45(4):e70155. doi: 10.1111/dar.70155 (PMC13077020; doi:10.1111/dar.70155)
Supplement: Supplementary file 1 — Data S1: Survey Measures. [file DAR-45-0-s001.docx]

**S1 – Survey Measures**

**Demographics**

What is your age?

What was your sex at birth?

| ❍ _1_ | Male |
| --- | --- |
| ❍ _2_ | Female |

What is your gender?

*Refers to current gender which may be different from sex assigned at birth and may be different from what is indicated on legal documents*

| ❍ _1_ | Man |
| --- | --- |
| ❍ _2_ | Woman |
| ❍ _3_ | Other gender (please specify): ______________ |
| ❑ _-8_ | Prefer not to say |

We know that people of different races do not have significantly different genetics. But our race still has important consequences, including how we are treated by different individuals and institutions. Which race category best describes you?

*Select all that apply*

| ❑ _1_ | Black (African, Afro-Caribbean, African Canadian descent) |
| --- | --- |
| ❑ _2_ | East/Southeast Asian (Chinese, Korean, Japanese, Taiwanese descent or Filipino, Vietnamese, Cambodian, Thai, Indonesian, other Southeast Asian descent) |
| ❑ _3_ | Indigenous (First Nations, Métis, Inuk/Inuit descent) |
| ❑ _4_ | Latino (Latin American, Hispanic descent) |
| ❑ _5_ | Middle Eastern (Arab, Persian, West Asian descent (e.g., Afghan, Egyptian, Iranian, Lebanese, Turkish, Kurdish)) |
| ❑ _6_ | South Asian (South Asian descent (e.g., East Indian, Pakistani, Bangladeshi, Sri Lankan, Indo-Caribbean) |
| ❑ _7_ | White (European descent) |
| ❑ _8_ | Other |
| ❑ _-8_ | Prefer not to say |

What is the **highest** certificate, diploma or degree that you have completed?

| ❍ _1_ | Less than high school diploma or its equivalent |
| --- | --- |
| ❍ _2_ | High school diploma or a high school equivalency certificate |
| ❍ _3_ | Trade certificate or diploma |
| ❍ _4_ | College, CEGEP, or other non-university certificate or diploma (other than trades certificates or diplomas) |
| ❍ _5_ | University certificate or diploma below the bachelor’s level |
| ❍ _6_ | Bachelor’s degree (e.g., B.A., B.Sc., LL.B.) |
| ❍ _7_ | University certificate, diploma or degree above the bachelor’s level |
| ❑ _-8_ | Prefer not to say |

*Note: This variable was recoded to 3 categories (High school or less/Unstated; Trades/college or non-university diploma; At least some university).*

What is your best estimate of your total **household income** received by all household members, from all sources, before taxes and deductions, during the year ending December 31, 2021?

| ❍ _1_ | Less than $10,000 |
| --- | --- |
| ❍ _2_ | $10,000 to less than $25,000 |
| ❍ _3_ | $25,000 to less than $50,000 |
| ❍ _4_ | $50,000 to less than $75,000 |
| ❍ _5_ | $75,000 to less than $100,000 |
| ❍ _6_ | $100,000 to less than $125,000 |
| ❍ _7_ | $125,000 to less than $150,000 |
| ❍ _8_ | $150,000 or more |
| ❑ _-8_ | Prefer not to say |

*Note: This variable was recoded to 4 categories (Less than $50,000; $50,000-$99,999; $100,000 or more; Unstated).*

**Past 12-month vape product use**

In the **past 12 months**, have you used the following cannabis products?

*Select all that apply.*

| ❑ _1_ | Dried flower/leaf |
| --- | --- |
| ❑ _2_ | Hashish/kief |
| ❑ _3_ | Cannabis oil for oral use – e.g., in dropper/syringe, softgel/capsules, spray bottle, tinctures |
| ❑ _4_ | Cannabis vape pens/cartridges |
| ❑ _5_ | Cannabis concentrate/extracts – e.g., shatter/wax/budder/butane honey oil/rosin |
| ❑ _6_ | Cannabis edible food products – e.g., cookies, candy |
| ❑ _7_ | Cannabis beverages – e.g., sparkling water, tea, soft drinks, dissolvable powder |
| ❑ _8_ | Topicals – e.g., lotion, ointment, creams applied to skin |
| ❑ _9_ | Other (please specify): _______________ |

**Vape product THC concentration**

2020-2022: When choosing cannabis products for **non-medical** purposes, what levels of THC and CBD do you typically use for:
 **Cannabis vape pens/cartridges**?

*Please make a selection in each of the CBD and THC sections.*

|  | THC Levels |
| --- | --- |
| ❑ _1_ | **High** (greater than 20% THC or greater than 200mg/g THC) |
| ❑ _2_ | **Moderate** (between 10% and 20% THC or 100mg/g to 200mg/g THC) |
| ❑ _3_ | **Low** (less than 10% THC or less than 100mg/g THC) |
| ❑ _4_ | **None** (0% THC or 0mg/g THC) |
| ❑ _5_ | Don't know/Not sure |
|  | CBD Levels |
| ❑ _6_ | **High** (greater than 20% CBD or greater than 200mg/g CBD) |
| ❑ _7_ | **Moderate** (between 10% and 20% CBD or 100mg/g to 200mg/g CBD) |
| ❑ _8_ | **Low** (less than 10% CBD or less than 100mg/g CBD) |
| ❑ _9_ | **None** (0% CBD or 0mg/g CBD) |
| ❑ _10_ | Don't know/Not sure |

2023: When choosing cannabis products, what levels of **THC** do you typically use for **cannabis vape pens/cartridges**?

*Please enter a number and select a unit from the drop down menu.*

Minimum: 0, Maximum: 1000

__________%

_________mg

_________mg/g

| ❑ _-8_ | Prefer not to say |
| --- | --- |
| ❑ _-9_ | Don't know |

**Vape product frequency of use**

In the **past 12 months**, how often have you used the following cannabis products?

| 1. | Dried flower/leaf |
| --- | --- |
| 2. | Hashish/kief |
| 3. | Cannabis oil for oral use – e.g., in dropper/syringe, softgel/capsules, spray bottle, tinctures |
| 4. | Cannabis vape pens/cartridges |
| 5. | Cannabis concentrate/extracts – e.g., shatter/wax/budder/butane honey oil/rosin |
| 6. | Cannabis edible food products – e.g., chocolate, baked goods, soft chews |
| 7. | Cannabis beverages – e.g., sparkling water, tea, soft drinks, dissolvable powder |
| 8. | Topicals – e.g., lotion/cream, ointment, bath products, patches |
| 9. | Other product |

| ❍ _1_ | Less than 1 day per month |
| --- | --- |
| ❍ _2_ | 1 day per month |
| ❍ _3_ | 2 or 3 days per month |
| ❍ _4_ | 1 or 2 day(s) per week |
| ❍ _5_ | 3 or 4 days per week |
| ❍ _6_ | 5 or 6 days per week |
| ❍ _7_ | Daily |

**ASSIST Questions**

During the **past 3 months**, how often did you use cannabis?

| ❍ _1_ | Never |
| --- | --- |
| ❍ _2_ | Once or twice |
| ❍ _3_ | Monthly |
| ❍ _4_ | Weekly |
| ❍ _5_ | Daily or almost daily |

During the **past 3 months**, how often have you had a strong desire or urge to use cannabis?

| ❍ _1_ | Never |
| --- | --- |
| ❍ _2_ | Once or twice |
| ❍ _3_ | Monthly |
| ❍ _4_ | Weekly |
| ❍ _5_ | Daily or almost daily |

During the **past 3 months**, how often has your use of cannabis led to health, social, legal or financial problems?

| ❍ _1_ | Never |
| --- | --- |
| ❍ _2_ | Once or twice |
| ❍ _3_ | Monthly |
| ❍ _4_ | Weekly |
| ❍ _5_ | Daily or almost daily |

During the **past 3 months**, how often have you failed to do what was normally expected of you because of your use of cannabis?

| ❍ _1_ | Never |
| --- | --- |
| ❍ _2_ | Once or twice |
| ❍ _3_ | Monthly |
| ❍ _4_ | Weekly |
| ❍ _5_ | Daily or almost daily |

Has a friend or relative or anyone else ever expressed concern about your use of cannabis?

| ❍ _1_ | No, never |
| --- | --- |
| ❍ _2_ | Yes, in the past 3 months |
| ❍ _3_ | Yes, but not in the past 3 months |

Have you ever tried and failed to control, cut down or stop using cannabis?

| ❍ _1_ | No, never |
| --- | --- |
| ❍ _2_ | Yes, in the past 3 months |
| ❍ _3_ | Yes, but not in the past 3 months |

**Cannabis co-use with tobacco or e-cigarette with nicotine**

During the **past 12 months**, when you used cannabis, how often did you combine it with any of the following substances?

*"Combine" means mixed or consumed at the same time.*

| 1. | Alcohol |
| --- | --- |
| 2. | Tobacco or e-cigarette with nicotine |
| 3. | Opioids (e.g., oxy, Dilaudid^®^, morphine, Demerol^®^, Tylenol #3^®^, heroin, fentanyl) |
| 4. | Stimulants (e.g., Ritalin^®^, Adderall^®^, cocaine, methamphetamine, ecstasy/MDMA) |
| 5. | Sedatives/tranquilizers (e.g., diazepam, lorazepam, Valium^®^, Ativan^®^, alprazolam, Xanax^®^, clonazepam, Rivotril^®^) |
| 6. | Hallucinogens/dissociatives (e.g., LSD, magic mushrooms, ketamine, PCP) |

| ❍ _1_ | Never |
| --- | --- |
| ❍ _2_ | Rarely |
| ❍ _3_ | Sometimes |
| ❍ _4_ | Often |
| ❍ _5_ | Always |
