# Supplementary material for: Exploring Age and Sex Differences in the Use of Cannabis Vaping Products: Results From the Canadian Cannabis Survey 2020–2023
Source: Drug Alcohol Rev. 2026 Apr 13;45(4):e70155. doi: 10.1111/dar.70155 (PMC13077020; doi:10.1111/dar.70155)
Supplement: Supplementary file 2 — Table S2: Sample characteristics of past 12‐month cannabis consumers aged 16 years and older, Canadian Cannabis Survey, 2020–2023. [file DAR-45-0-s003.docx]

Table S2. Sample characteristics of past 12-month cannabis consumers^1^ aged 16 years and older, Canadian Cannabis Survey, 2020-2023

| **Characteristic** | **2020 (n=3405)**  **% (n)** | **2021 (n=2802)**  **% (n)** | **2022 (n=2801)**  **% (n)** | **2023 (n=2972)**  **% (n)** |
| --- | --- | --- | --- | --- |
| **Sex at birth** |  |  |  |  |
| Female | 44.5^↑^ (1539) | 45.2 (1369) | 45.7 (1305) | 45.9 (1408) |
| Male | 55.5^↓^ (1866) | 54.8 (1433) | 54.3 (1496) | 54.1 (1564) |
| **Gender identity** |  |  |  |  |
| Man | 54.8 (1846) | 53.3 (1323) | 52.9 (1453) | 52.8 (1520) |
| Woman | 43.8 (1505) | 43.9 (1323) | 42.9 (1225) | 43.6 (1330) |
| Other gender identity | 0.9 (34)^E^ | 1.4 (45) | 2.5^↑^(68) | 2.3 (77) |
| Unstated | 0.5 (20) ^E^ | 1.4 (42)^E^ | 1.8 (55) | 1.4 (45) |
| **Age group, years** |  |  |  |  |
| 16-19 | 9.0 (377) | 8.3 (339) | 7.1 (293) | 8.8 (295) |
| 20-24 | 1.5 (933) | 1.5^↓^ (554) | 1.4 (561) | 1.3 (635) |
| 25-34 | 25.9 (775) | 24.2 (529) | 25.9 (555) | 25.9 (534) |
| 35-44 | 17.7 (509) | 18.8 (564) | 18.7 (581) | 18.5^↓^ (537) |
| 45-54 | 13.4 (381) | 14.1 (358) | 12.8 (371) | 12.2 (414) |
| 55+ | 18.8 (430) | 19.7 (458) | 21.8 (440) | 21.1 (557) |
| **Ethnicity** |  |  |  |  |
| White | 78.6 (7980) | 77.3 (7446) | 75.7 (7002) | 76.1 (8397) |
| Another ethnicity/mixed/unstated | 21.4 (2841) | 22.7 (3287) | 24.3 (3046) | 23.9 (3293) |
| **Highest education level** |  |  |  |  |
| High school or less/unstated | 29.2 (1124) | 28.8 (924) | 28.4 (896) | 28.9 (951) |
| Trades/college or non-university diploma | 30.9 (1042) | 32.4 (869) | 31.2 (857) | 26.9 (809) |
| At least some university | 39.9 (1239) | 38.7 (1009) | 40.4 (1048) | 44.2 (1212) |
| **Household income** |  |  |  |  |
| Less than $50,000 | 24.2 (930) | 24.3 (708) | 21.3 (635) | 19.0 (600) |
| $50,000-$99,999 | 31.7 (1029) | 32.1 (879) | 32.4 (870) | 30.0 (872) |
| $100,000 or more | 36.9 (1179) | 35.9 (990) | 40.4 (1113) | 43.8 (1265) |
| Unstated | 7.2 (267) | 7.7 (225) | 5.9 (183) | 7.2 (235) |

^1^ Sample excludes those who consumed cannabis exclusively for medical purposes and those with missing data on vaping. ^↑^Indicates value should be rounded up if reported as a whole number. ^↓^Indicates value should be rounded down if reported as a whole number. ^E^ Interpret with caution due to moderate sampling variability.
