# Supplementary material for: Exploring Age and Sex Differences in the Use of Cannabis Vaping Products: Results From the Canadian Cannabis Survey 2020–2023
Source: Drug Alcohol Rev. 2026 Apr 13;45(4):e70155. doi: 10.1111/dar.70155 (PMC13077020; doi:10.1111/dar.70155)
Supplement: Supplementary file 3 — Table S3: Use of cannabis vaping products among past 12‐month cannabis consumers overall and by sex and age, 2020–2023. [file DAR-45-0-s002.docx]

**Table S3. Use of cannabis vaping products among past 12-month cannabis consumers overall and by sex and age, 2020-2023**

|  | **2020 (n=3405)** | **2021 (n=2802)** | **2022 (n=2801)** | **2023 (n=2972)** | **Odds of using vaping products in 2023 vs. 2020** *(reference year)* | **Differences among subgroups** |
| --- | --- | --- | --- | --- | --- | --- |
|  | **% (95% CI)** | **% (95% CI)** | **% (95% CI)** | **% (95% CI)** | **AOR (95% CI) ^a^** |  |
| **Any use of cannabis vaping products in past 12 months** |  |  |  |  |  |  |
| **Overall** | 21.7 (20.1, 23.2) | 29.0 (27.2, 30.9) | 33.3 (31.4, 35.3) | 36.8 (34.8, 38.7) | 2.19 (1.93, 2.49), p<0.001 | - |
| **Sex** |  |  |  |  | **AOR (95% CI) ^b^** | **AOR (95% CI) ^d^** |
| Female | 19.9 (17.6, 22.2) | 29.9 (27.2, 32.6) | 34.7 (31.8, 37.6) | 38.7 (35.8, 41.6) | 2.63 (2.17, 3.20), p<0.001 | *Reference* |
| Male | 23.1 (21.0, 25.3) | 28.2 (25.8, 30.8) | 32.2 (29.5, 34.8) | 35.1 (32.6, 37.7) | 1.89 (1.59, 2.24), p<0.001 | 0.97 (0.88, 1.06), p=0.491 |
| **Age** |  |  |  |  | **AOR (95% CI) ^c^** | **AOR (95% CI)^e^** |
| 16-19 | 32.5 (27.4, 37.6) | 50.8 (45.2, 56.4) | 59.9 (54.1, 65.7) | 66.0 (60.2, 71.7) | 4.00 (2.82, 5.67), p<0.001 | *Reference* |
| 20-24 | 27.5 (24.5, 30.5) | 40.1 (35.8, 44.5) | 47.6 (43.3, 51.9) | 54.1 (50.0, 58.1) | 3.11 (2.49, 3.89), p<0.001 | 0.72 (0.62, 0.84), p<0.001 |
| 25+ | 19.2 (17.4, 21.1) | 24.5 (22.4, 26.6) | 28.5 (26.2, 30.7) | 30.5 (28.3, 32.7) | 1.84 (1.57, 2.16), p<0.001 | 0.33 (0.29, 0.38), p<0.001 |
| ***Sensitivity analyses: results among adults 25+*** |  |  |  |  |  | **AOR (95% CI) ^e^**  *Reference=16-19 years ^f^* |
| 25-34 | 24.6 (21.4, 28.1) | 32.7 (28.6, 37.2) | 42.1 (37.6, 46.7) | 39.9 (35.6, 44.4) | 2.07 (1.60, 2.69), p<0.001 | 0.56 (0.47, 0.67), p<0.001 |
| 35-44 | 24.5 (20.6, 29.0) | 26.1 (22.2, 30.4) | 25.2 (21.5, 29.3) | 34.6 (30.4, 39.1) | 1.62 (1.19, 2.19), p=0.002 | 0.38 (0.32, 0.45), p<0.001 |
| 45-54 | 15.5 (11.9, 20.0) | 20.3 (16.1, 25.2) | 27.3 (22.5, 32.7) | 25.4 (21.0, 30.3) | 1.85 (1.24, 2.75), p=0.002 | 0.28 (0.23, 0.34), p<0.001 |
| 55+ | 9.5 (6.8, 13.1) | 15.9 (12.7, 19.9) | 15.7 (12.4, 19.7) | 18.3 (15.0, 22.0) | 2.09 (1.36, 3.22), p=0.001 | 0.17 (0.14, 0.21), p<0.001 |
| **Daily/almost daily use of cannabis vaping products** |  |  |  |  |  |  |
| **Overall** | 1.7 (1.2, 2.2) | 4.0 (3.2, 4.8) | 6.5 (5.4, 7.5) | 6.2 (5.2, 7.1) | 3.95 (2.82, 5.52), p<0.001 | - |
| **Sex** |  |  |  |  | **AOR (95% CI) ^b^** | **AOR (95% CI) ^d^** |
| Female | 1.5* (0.9, 2.2) | 4.1 (2.9, 5.2) | 6.9 (5.3, 8.5) | 5.9 (4.5, 7.3) | 4.12 (2.44, 6.96), p<0.001 | *Reference* |
| Male | 1.8* (1.2, 2.5) | 4.0 (2.9, 5.1) | 6.1 (4.7, 7.5) | 6.4 (5.1, 7.7) | 3.84 (2.47, 5.98), p<0.001 | 0.99 (0.82, 1.21), p=0.950 |
| **Age** |  |  |  |  | **AOR (95% CI) ^c^** | **AOR (95% CI) ^e^** |
| 16-19 | 3.1* (1.2, 5.0) | 6.2* (3.6, 7.6) | 9.3* (5.9, 12.6) | 9.6* (6.4, 11.3) | 3.33 (1.55, 7.16), p=0.002 | *Reference* |
| 20-24 | 1.9* (1.0, 2.8) | 5.6* (3.5, 7.6) | 8.8 (6.4, 11.3) | 6.9 (4.9, 8.9) | 3.78 (2.10, 6.80), p<0.001 | 1.00 (0.73, 1.36), p=0.986 |
| 25 and older | 1.5* (0.9, 2.1) | 3.5 (2.6, 4.4) | 5.8 (4.6, 7.0) | 5.6 (4.6, 6.7) | 4.06 (2.57, 6.26), p<0.001 | 0.80 (0.59, 1.09), p<0.001 |

^a^ Model adjusted for age, sex, education, income, ethnicity.

^b^ Model adjusted for age, education, income, ethnicity.

^c^ Model adjusted for sex, education, income, ethnicity.

^d^ Model adjusted for age, education, income, ethnicity, survey year (2020, 2021, 2022, 2023).

^e^ Model adjusted for sex, education, income, ethnicity, survey year (2020, 2021, 2022, 2023).

^f^ Results for 16-19 and 20-24 not shown to avoid duplicating previous rows.

AOR, adjusted odds ratio; CI, confidence interval.
